# Supplementary material for: A Pilot Study towards the Impact of Type 2 Diabetes on the Expression and Activities of Drug Metabolizing Enzymes and Transporters in Human Duodenum
Source: Int J Mol Sci. 2019 Jul 2;20(13):3257. doi: 10.3390/ijms20133257 (PMC6651059; doi:10.3390/ijms20133257)
Supplement: Supplementary file 1 [file ijms-20-03257-s001.pdf]

Appendix I: Method details

Quantification of Intestinal CYP450 mRNAs. For each participant, two biopsies (≈10 mg of tissues) were homogenized in 1 mL of TRIzol® and incubated for 5 min at room temperature. Chloroform (200 µl) was added, the mixture shaken for 15 sec and then, centrifuged at 16,000 g for 30 min at 4°C. The aqueous supernatant (500 µl) was transferred and ethanol 70% was added (1:1 v/v). RNA was extracted using the Qiagen kit (RNeasy Mini kit; Qiagen Sciences, MD, USA) according to the manufacturer's recommendations. RNA concentration and quality were assessed by spectrometry. Total RNA (2 µg) from each sample was used for reverse transcription as described previously and resulting cDNA was kept at -80°C until use.[12] Real-time quantitative PCR was performed using TaqMan® probe and primer sets from Applied Biosystem (Foster, CA, USA) on a QuantStudio 6 Flex System (Life Technologies Inc., Burlington, ON, Canada) as detailed elsewhere.[12] The analysed drug metabolizing enzymes and transporters were: CYP1A1 (Hs01054796_g1), CYP2B6 (Hs04183483_g1), CYP2C8 (Hs00946140_g1), CYP2C9 (Hs00426397_m1), CYP2C19 (Hs00426380_m1), CYP2D6 (Hs02576167_m1), CYP2E1 (Hs00559368_m1), CYP2J2 (Hs00951113_m1), CYP3A4 (Hs00604506_m1), CYP3A5 (Hs01070905_m1) CES-1 (Hs00275607_m1), CES-2 (Hs01077945_m1) ABCB1 (Hs00184500)_m1) ABCG2 (Hs01053790_m1) OATP2B1 (Hs01030343_m1). GAPDH (Mm99999915_g1) and NUP214 (Hs01090093_m1) were used as housekeeping genes and villin (Hs00200229_m1) as a calibrator. The relative quantification of various gene expression was calculated with the comparative CT method using the formula $2^{-\Delta CT}$.[38] All measured mRNA levels were within the quantifiable range (CT values <35). In addition, mRNA levels associated with the expression of each CYP450 isoenzymes between our two study groups were determined using a calibrator and the following formula: $2^{-\Delta\Delta CT}$.[38,39]

CYP450 genotype analysis. Blood samples for genotyping were kept at room temperature and DNA was extracted from leukocytes according to standard procedures within seven days using the GenElute™ Blood Genomic DNA kit (Sigma Aldrich, Oakville, Can). Resulting purified genomic DNAs were stored at -20°C until genotyping procedures for major CYP450 isoforms were performed. Variants for the isoenzymes *CYP2B6*, *CYP2C9*, *CYP2J2* and *CYP3A4/5* were detected using the Taqman® qRT-PCR SNP Genotyping Assay (Life Technologies, Burlington, Can). The PCR assay was performed and analysed using the QuantStudio™ 6 Flex System (Life Technologies, Burlington, Can). The SNP Genotyping assay was completed using specific probes for all SNPs (Life Technologies, Burlington, Can). Tested SNPs for *CYP2B6* were **5* (rs3211371) and **9* (rs3745274) using specific probes C_30634242_40 and C_7817765_60, respectively. Two SNPs for *CYP2C9* were considered using specific probes: *CYP2C9*2* (rs1799853; C_25625805_10) and *CYP2C9*3* (rs1057910; C_27104892_10). Presence of *CYP2J2*6* (rs72547598) and *CYP2J2*7* (rs890293) were verified using specific probes C_27859821_10 and C_9581699_80. The tested SNP for the isoform *CYP3A4* was *CYP3A4*22* (rs35599367) with specific probe C_59013445_10. For *CYP3A5*, presence of two SNPs were tested; *CYP3A5*3* (rs776746) and *CYP3A5*6* (rs10264272). The specific probes used were C_26201809_30 and C_30203950_10, respectively.

Appendix II:

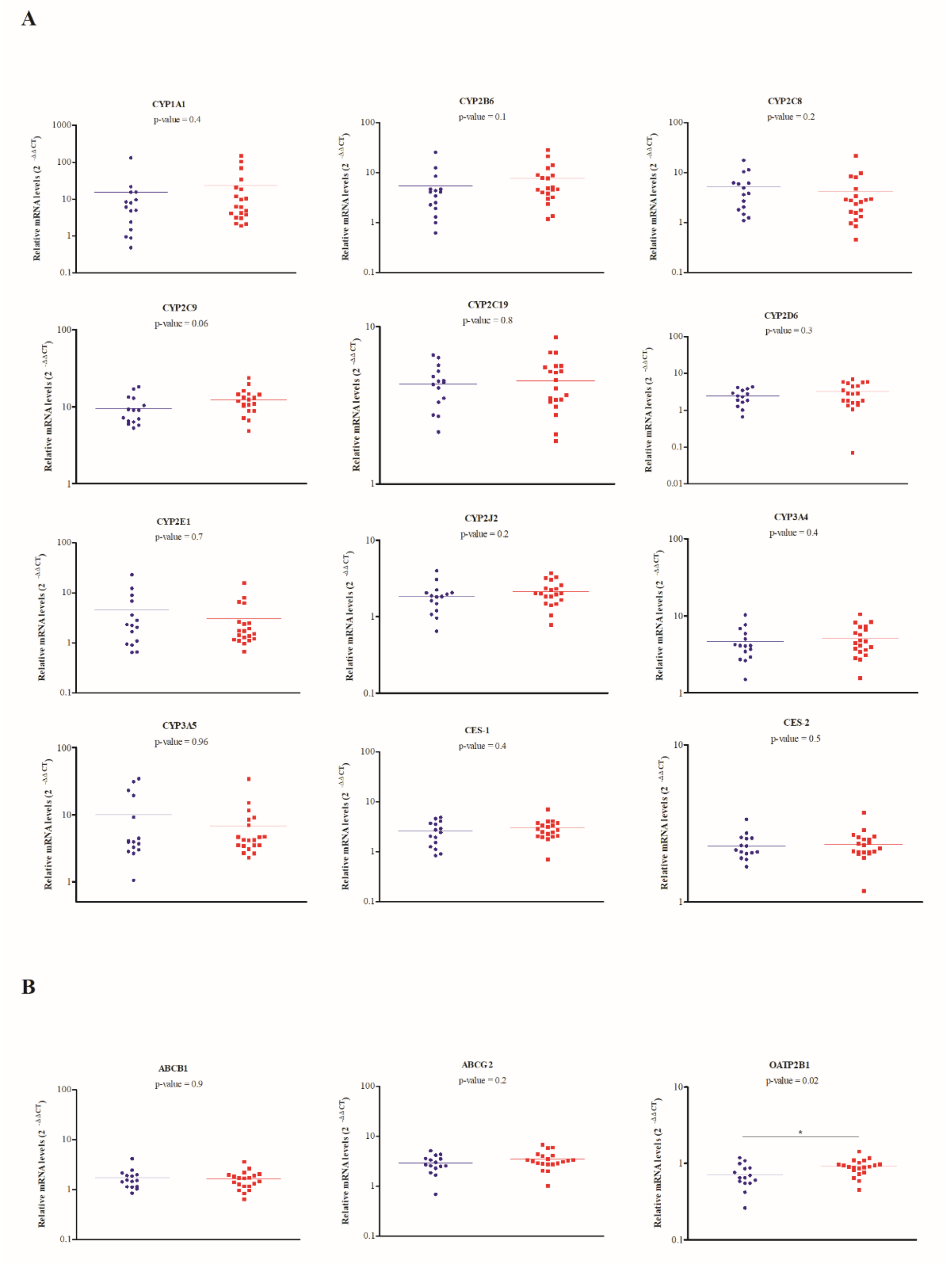

Figure S1. Quantified mRNA transcript levels expressed as N-fold differences relative to the average expression of housekeeping genes and calibrator ($2^{-\Delta\Delta CT}$) for (**A**) all drug metabolizing enzyme proteins and (**B**) all drug transporters in patients with T2D (red squares ■) versus non-diabetic controls (blue circles ●). Each experiment was performed three times in triplicates, means are displayed for all individuals and Mann Whitney t-test was performed on overall mean values.

Appendix III:

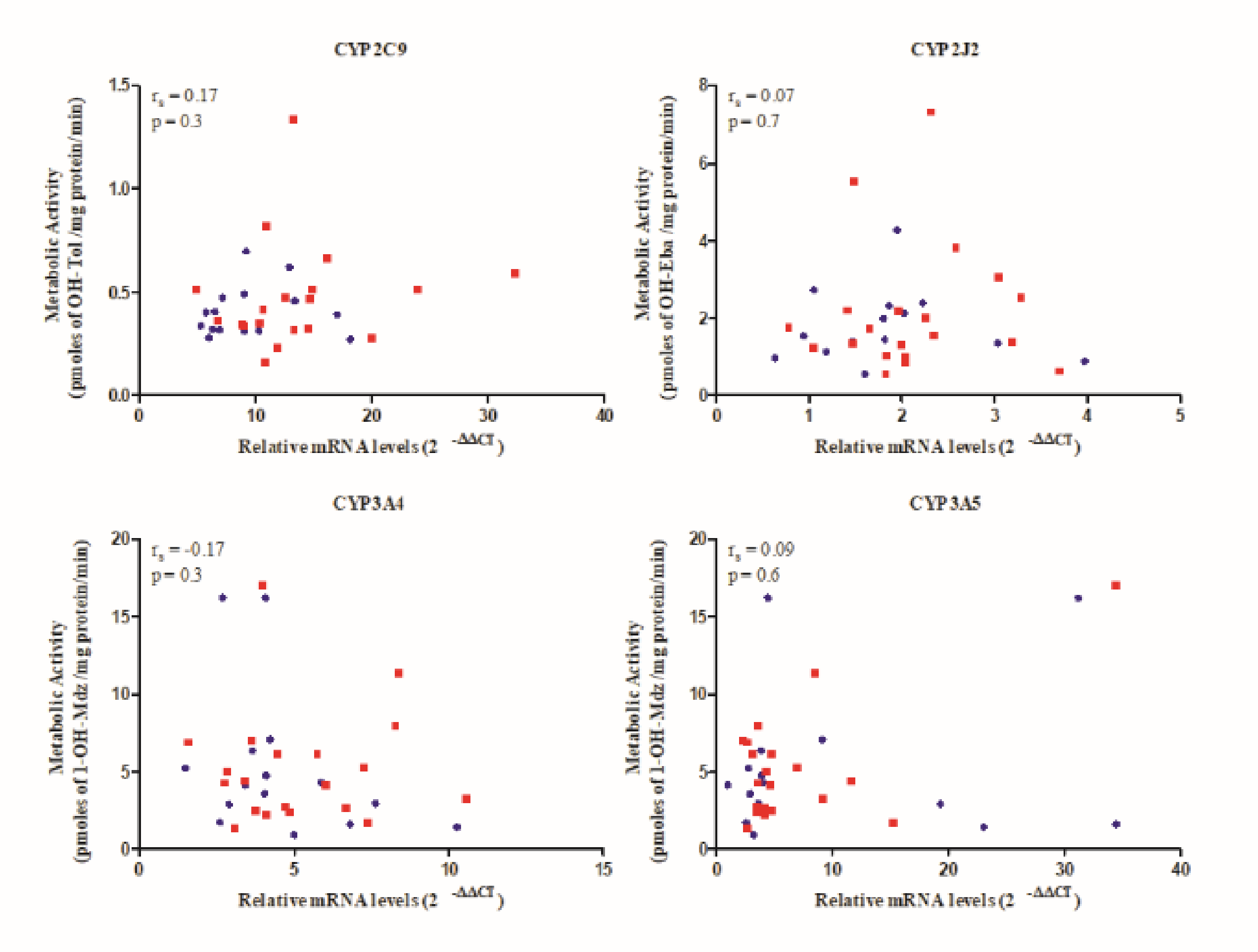

Figure S2. Correlations between mRNA expression levels as N-fold differences relative to the average expression of housekeeping genes and calibrator ($2^{-\Delta\Delta CT}$) and metabolic activities expressed as metabolite formation rates (pmoles mg $protein^{-1}$ min^{-1}) in both study groups for CYP2C9 (OH-Tol), CYP2J2 (OH-Eba), CYP3A4 and CYP3A5 (1-OH-Mdz). Patients with T2D are displayed with red squares (■) and non-diabetic controls are blue circles (●). Spearman's rank correlation coefficient (r_s) and respective p-values are provided on each graph.

Appendix IV:

Table S1. Correlation between proinflammatory cytokine levels and CYP450 activities

Isozymes	Proinflammatory cytokines	Correlation coefficient (r_s)	p-value
CYP2C9	IFN-γ	- 0.09	0.6
	IL-1β	- 0.06	0.7
	IL-6	0.10	0.6
	TNF-α	0.12	0.5
CYP2J2	IFN-γ	- 0.10	0.6
	IL-1β	0.21	0.2
	IL-6	- 0.04	0.8
	TNF-α	0.01	0.9
CYP3A	IFN-γ	- 0.19	0.3
	IL-1β	0.10	0.6
	IL-6	- 0.19	0.3
	TNF-α	0.05	0.8

r_s, Spearman's rank correlation coefficient; IFN-γ, interferon-gamma; IL-1β, interleukine-1 beta; IL-6, interleukine-6; TNF-α, tumour necrosis factor alpha

Appendix V:

Table S2. Correlation between CYP450 activities and T2D-related and demographic covariables.

Isozymes	Covariables	Correlation coefficient (r_s)	p-value
CYP2C9	Glycemia	0.32	0.06
	HbA1c	0.35	0.04
	HOMA-IR	0.07	0.67
	Insulinemia	0.02	0.90
	Age	0.14	0.43
	BMI	0.12	0.48
CYP2J2	Glycemia	0.03	0.86
	HbA1c	0.05	0.77
	HOMA-IR	0.09	0.59
	Insulinemia	0.13	0.44
	Age	0.01	0.97
	BMI	- 0.07	0.67
CYP3A	Glycemia	0.18	0.30
	HbA1c	0.12	0.50
	HOMA-IR	0.24	0.15
	Insulinemia	0.29	0.09
	Age	- 0.04	0.82
	BMI	- 0.12	0.47

r_s; Spearman's rank correlation coefficient

Correlation of glycemia (mmol L^{-1}), HbA1c (% glycated haemoglobin), HOMA-IR (homeostatic model assessment of insulin resistance), insulinemia (pmol L^{-1}), Age (years) and BMI (body mass index in kg $m^{2\ -1}$) with CYP450s metabolic activity (rates of pathway-specific metabolite formation in pmoles mg $protein^{-1}$ min^{-1}) for CYP2C9 (Tolbutamide → Hydroxytolbutamide), CYP2J2 (Ebastine → Hydroxyebastine) and CYP3A (Midazolam → 1'-Hydroxymidazolam)

Appendix VI:

Table S3. Influence of time since diagnostic of T2D on metabolic activity of CYP2C9, CYP2J2 and CYP3A.

**Time since diagnostic** (years)	**Metabolic activity** (pmoles mg protein^{-1} min^{-1})	**p-value**
CYP2C9		
< 5 (n=4)	0.37 ± 0.10	
5 to 10 (n=6)	0.47 ± 0.20	0.5
> 10 (n=9)	0.53 ± 0.34	
CYP2J2		
< 5 (n=4)	1.41 ± 0.63	
5 to 10 (n=7)	2.63 ± 2.20	0.5
> 10 (n=9)	2.13 ± 1.61	
CYP3A		
< 5 (n=4)	3.75 ± 1.61	
5 to 10 (n=7)	5.43 ± 5.32	0.4
> 10 (n=9)	5.79 ± 3.03	

Values of metabolic activities are presented as mean ± SD
p-values for Kruskall-Wallis test are reported
Influence of time since diagnostic (years) on CYP450s metabolic activity (rates of pathway-specific metabolite formation in pmoles mg protein^{-1} min^{-1}) for CYP2C9 (Tolbutamide → Hydroxytolbutamide), CYP2J2 (Ebastine → Hydroxyebastine) and CYP3A (Midazolam → 1'-Hydroxymidazolam)

Appendix VII:

Table S4. Qualitative analysis of the influence of genetic variants on metabolic activity in overall study population.

Genetic Variant	Metabolic activity (pmoles mg protein^{-1} min^{-1})
CYP2C9	
*CYP2C9 *1/*1* (n=24)	0.46 ± 0.24
*CYP2C9 *1/*2* (n=6)	0.47 ± 0.17
*CYP2C9 *1/*3* (n=3)	0.38 ± 0.09
*CYP2C9 *2/*2* and **3/*3* (n=2)	0.32 ± 0.06
CYP2J2	
*CYP2J2 *1/*1* (n=32)	2.07 ± 1.45
*CYP2J2 *1/*7* (n=4)	1.04 ± 0.40
CYP3A4	
*CYP3A4 *1/*1* (n=33)	5.35 ± 4.28
*CYP3A4 *1/*22* (n=3)	2.97 ± 0.32
CYP3A5	
Expressers: **1/*1, *1/*3* and **1/*6* (n=6)	6.01 ± 5.38
Non-expressers: **3/*3* (n=30)	4.98 ± 3.95

No statistical analysis was performed due to the small number of subjects with variant alleles.

Reported metabolic activities are rates for pathway-specific metabolite formation (pmoles mg protein^{-1} min^{-1}) for CYP2C9 (Tolbutamide → Hydroxytolbutamide), CYP2J2 (Ebastine → Hydroxyebastine) and CYP3A (Midazolam → 1'-Hydroxymidazolam)
